# Variations in Hypoxia Impairs Muscle Oxygenation and Performance during Simulated Team-Sport Running

**DOI:** 10.3389/fphys.2017.00080

**Published:** 2017-02-10

**Authors:** Alice J. Sweeting, François Billaut, Matthew C. Varley, Ramón F. Rodriguez, William G. Hopkins, Robert J. Aughey

**Affiliations:** ^1^Institute of Sport, Exercise and Active Living, Victoria UniversityMelbourne, VIC, Australia; ^2^Département de Kinesiology, Université LavalQuébec, QC, Canada

**Keywords:** altitude, non-motorized treadmill, near-infrared spectroscopy, repeated sprints

## Abstract

**Purpose:** To quantify the effect of acute hypoxia on muscle oxygenation and power during simulated team-sport running.

**Methods:** Seven individuals performed repeated and single sprint efforts, embedded in a simulated team-sport running protocol, on a non-motorized treadmill in normoxia (sea-level), and acute normobaric hypoxia (simulated altitudes of 2,000 and 3,000 m). Mean and peak power was quantified during all sprints and repeated sprints. Mean total work, heart rate, blood oxygen saturation, and quadriceps muscle deoxyhaemoglobin concentration (assessed via near-infrared spectroscopy) were measured over the entire protocol. A linear mixed model was used to estimate performance and physiological effects across each half of the protocol. Changes were expressed in standardized units for assessment of magnitude. Uncertainty in the changes was expressed as a 90% confidence interval and interpreted via non-clinical magnitude-based inference.

**Results:** Mean total work was reduced at 2,000 m (−10%, 90% confidence limits ±6%) and 3,000 m (−15%, ±5%) compared with sea-level. Mean heart rate was reduced at 3,000 m compared with 2,000 m (−3, ±3 min^−1^) and sea-level (−3, ±3 min^−1^). Blood oxygen saturation was lower at 2,000 m (−8, ±3%) and 3,000 m (−15, ±2%) compared with sea-level. Sprint mean power across the entire protocol was reduced at 3,000 m compared with 2,000 m (−12%, ±3%) and sea-level (−14%, ±4%). In the second half of the protocol, sprint mean power was reduced at 3,000 m compared to 2,000 m (−6%, ±4%). Sprint mean peak power across the entire protocol was lowered at 2,000 m (−10%, ±6%) and 3,000 m (−16%, ±6%) compared with sea-level. During repeated sprints, mean peak power was lower at 2,000 m (−8%, ±7%) and 3,000 m (−8%, ±7%) compared with sea-level. In the second half of the protocol, repeated sprint mean power was reduced at 3,000 m compared to 2,000 m (−7%, ±5%) and sea-level (−9%, ±5%). Quadriceps muscle deoxyhaemoglobin concentration was lowered at 3,000 m compared to 2,000 m (−10, ±12%) and sea-level (−11, ±12%).

**Conclusions:** Simulated team-sport running is impaired at 3,000 m compared to 2,000 m and sea-level, likely due to a higher muscle deoxygenation.

## Introduction

Athletic performance is reduced at altitude, including elevations of 580 m (Gore et al., 1997). Team-sport matches are contested at a range of terrestrial altitudes including 1,600 m (Johannesburg, South Africa) and 3,600 m (La Paz, Bolivia). The impact of moderate to high altitude on junior-elite soccer performance has recently been investigated (Aughey et al., 2013; Garvican et al., 2013; Buchheit et al., 2015). At 1,600 m, total distance and high-speed running were reduced by 9 and 15%, respectively (Garvican et al., 2013). At 3,600 m, the 5-min peak period of high-speed running and total distance covered was lowered by 20.8 and 9.1%, respectively (Aughey et al., 2013). Acclimatization to, and native residence at, 3,600 m does not protect against the detrimental effects of altitude on team-sport athlete match running (Aughey et al., 2013). Altitude therefore has a substantial impact on running performance during junior-elite soccer matches. Understanding the magnitude and impact of altitude exposure on team-sport match running may assist in optimizing athlete physical preparation for playing at altitude.

The influence of altitude on team-sport athlete match running is typically investigated through repeated sprint laboratory studies. The capacity to perform sprint efforts is considered important for scoring during team-sport matches (Faude et al., 2012). Laboratory repeated sprint tests, performed on a non-motorized treadmill, are generally designed to replicate periods of match running and allow measurement of an athlete's capacity to resist fatigue. In team-sport athletes, peak power during three sets of nine repeated sprints, of 4-s in duration, was maintained at a simulated altitude of 3,000 m but not 4,000 m (Goods et al., 2014). The peak speed performed by untrained individuals is reduced during 10 6-s repeated-sprints in varying hypoxic conditions compared to normoxia (Bowtell et al., 2014). The distance covered by amateur team-sport athletes during the final two sets of four 4-s repeated sprints was also reduced in normobaric hypoxia compared to normoxia (Morrison et al., 2015). Simulated altitude therefore has a detrimental effect on laboratory repeated sprint performance. However, assessing the impact of titrated altitude during controlled laboratory studies does not replicate the chaotic and complex nature of team-sport matches (Sirotic and Coutts, 2007). During repeated sprint laboratory studies, with known exercise and rest periods, participants may plan their physical output. In matches, team-sport athletes perform low-speed activity interspersed with high-intensity movement. Exercise intensity may be regulated dependent upon an individual's ability to perform high-intensity activity (Castagna and Abt, 2003). Under conditions of environmental stress, athletes may moderate this low-speed activity to preserve the capacity to perform hard efforts, including repeated sprints (Aughey et al., 2013, 2014). Laboratory studies designed to mimic environmental stress, such as simulating altitude, should therefore include periods of lower-intensity work interspersed with repeated sprint efforts to simulate the running performed by team-sport athletes during matches.

The impact of altitude on physical performance during a simulated team-sport movement protocol was recently examined (Aldous et al., 2015). Total and high-speed distance covered by team-sport athletes during a 90-min protocol was reduced by 4% at 1,000 m simulated altitude compared to sea-level. The distance covered in each of the two 45-min halves was 10% lower at altitude compared to sea-level, with total sprint distance also reduced in the final 15 min of the protocol. Perceived exertion across the entire protocol was also 7% lower at sea-level compared to altitude. Since team-sport matches are contested at a range of altitudes around the world, the dose-response relationship of varying altitudes on simulated match running should be explored. The subsequent impact of these altitudes on the physiological determinants of team-sport running should also be examined.

Physiological determinants including neural, ionic and metabolic factors underpin the capacity to perform repeated sprint efforts (Billaut et al., 2012). Moderately trained individuals with a high maximal oxygen uptake ($\dot{\text{V}}$O_2max_) may have an increased resynthesis rate of phosphocreatine (PCr), a vital substrate during intermittent high-intensity exercise (Harris et al., 1976). Attained exclusively via aerobic sources, the resynthesis of PCr is dependent upon O_2_ and can be completely suppressed via limb blood flow occlusion (Harris et al., 1976). Muscle O_2_ kinetics and PCr resynthesis are also tightly linked (Kime et al., 2003). A high perfusion and reoxygenation rate of the active musculature during recovery periods, assessed via near-infrared spectroscopy (NIRS), is paramount to reproduce a high performance in a subsequent bout (Ufland et al., 2012). The delivery of O_2_ is also highly sensitive to manipulations of environmental O_2_ and compromised at altitude, which may contribute to impairment during repeated-sprint exercise (Billaut and Buchheit, 2013). The capacity of skeletal muscle to be reoxygenated during 30 s of passive rest between repeated sprint running efforts is reduced by up to 33% in hypoxia (Bowtell et al., 2014), although NIRS has not been utilized to assess muscle reoxygenation during simulated team-sport running at varying altitudes. Since PCr resynthesis is a main factor of performance during repeated sprint efforts, the slower repletion of this substrate at altitude may be detrimental to team-sport running performance. Therefore, the aim of the present study was to quantify the effect of acute, titrated hypoxia on physical output and physiological responses during simulated team-sport running.

## Methods

### Participants

Seven (six males and one female) individuals (age 27.0 ± 6.6 years; height 179.7 ± 6.1 cm; $\dot{\text{V}}$O_2max_ 59.5 ± 5.1 mL.kg^−1^.min^−1^; body mass 75.1 ± 10.2 kg at commencement of study, [mean ± Standard Deviation (SD)] provided written informed consent to participate in the study. Participants were from team-sport plus endurance backgrounds and trained at least twice per week. The study was approved by the university Human Research Ethics Committee and conformed to the declaration of Helsinki. Participants were sea-level natives and had not visited >2,000 m altitude for more than 24 h in the 4 months prior to participating in the study.

### Study design

A single-blind randomized design, with altitude counter-balanced, was employed. All testing was conducted within a 23.92 m^2^ environmental exercise laboratory. During their first visit, participants completed a $\dot{\text{V}}$O_2max_ test to assess maximal aerobic power. The test consisted of 3 × 4 min periods of incremental running before speed was maintained and gradient increased by 1% each minute until volitional exhaustion (Robertson et al., 2010). Participants then returned for a separate familiarization session, involving a 5 min warm-up at 100 W on a cycle ergometer (Velotron, Seattle, USA) followed by four maximal 4 s sprints, each separated by 14 s of passive recovery, on the non-motorized treadmill (Woodway Force, Waukesha, WI, USA). Individual speed ranges for the simulated team-sport movement protocol, described below, were established from the maximal speed attained during six sprint efforts on the non-motorized treadmill (Sirotic and Coutts, 2008). Participants then completed a modified (condensed to 13 min) version of the team-sport running protocol. The familiarization session, 1 week after the preliminary visit, involved completion of the entire simulated team-sport movement protocol and NIRS measurements. Participants then completed a testing session at sea-level followed by two randomized, counter-balanced sessions under titrated hypoxic conditions. Each session involved a standardized 5 min cycling warm-up at 100 W and completion of the simulated team-sport running protocol. Normobaric hypoxia was created through nitrogen injection into the environmental exercise laboratory. The F_i_O_2_ was ~0.163 and ~0.143, simulating altitudes of 2,000 m and 3,000 m, respectively. Simulated altitudes were based on the definition of low to moderate altitude (Bartsch et al., 2008). Each session was conducted at similar times of the day for each participant to limit influence of the circadian rhythm on sprint-induced neuromuscular fatigue (Giacomoni et al., 2006). All sessions were separated by 1 week. Participants were encouraged to refrain from physical activity and caffeine in the 24 h prior to each session.

### Simulated team-sport running protocol

Participants were secured onto the non-motorized treadmill using a manufacturer provided belt. The belt connected to a horizontal force transducer, adjusted for each participant by measuring and reproducing the displacement between the anterior superior illiac spine and the belt, as per Serpiello et al. (2011). Participants were encouraged to keep this consistent throughout all sessions. The force transducers were calibrated, before each participant's session, according to the manufacturer's guidelines.

**Figure 1:**
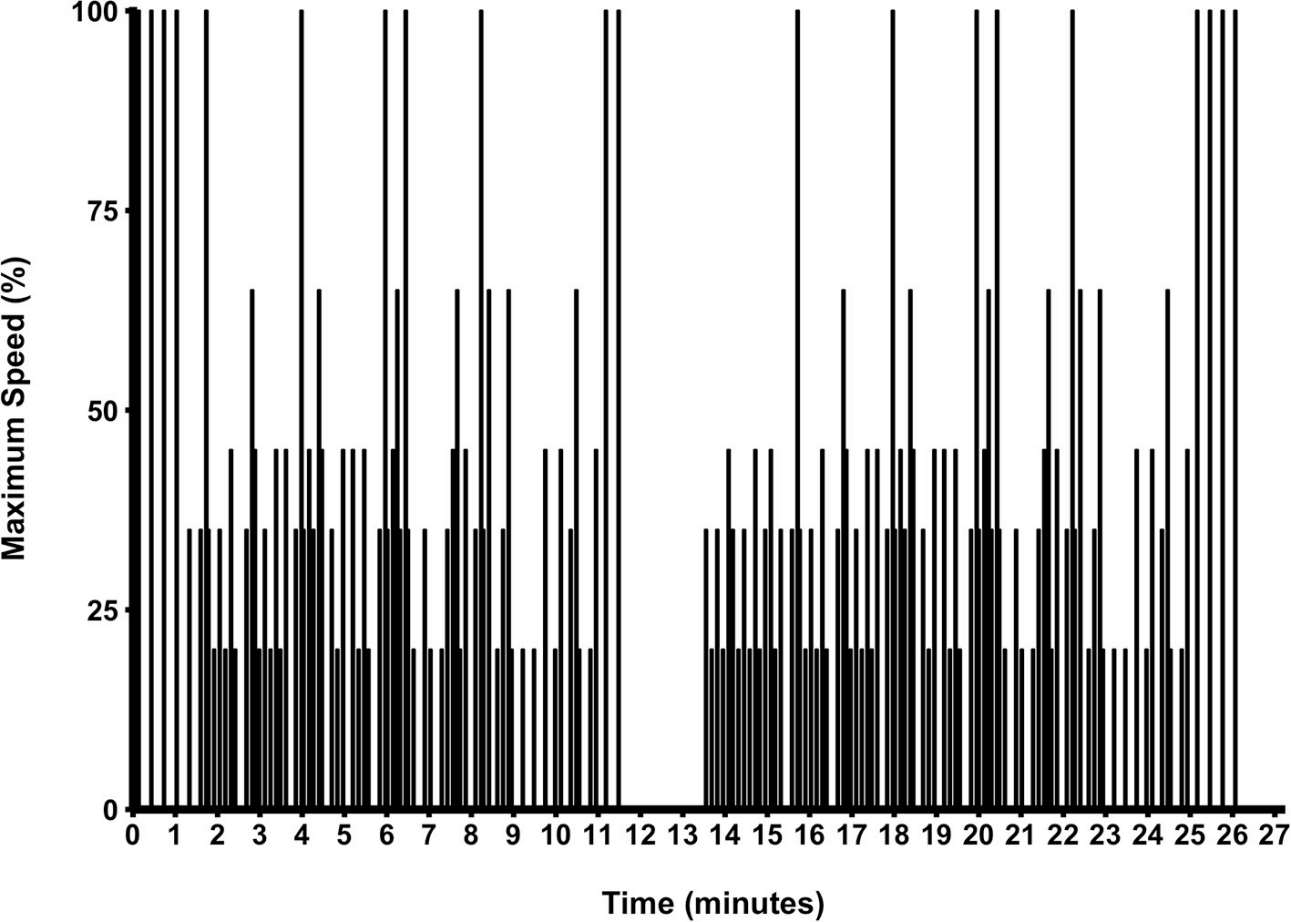
**The simulated team-sport running protocol**.

The 26.4 min protocol (Zois et al., 2013) required participants to move through six individually-established speed zones, displayed in Figure 1. The total percentage of each activity was; standing (33%), walking (23%), jogging (23%), running (13%), fast running (4%), and sprinting (4%). The protocol included three repeated-sprint tests (set 1 and 3; four 4 s sprints and set 2; two 4 s sprints, all interspersed with 14 s passive recovery) and 10 single sprint efforts, all 4 s in duration. Visual (screen above the non-motorized treadmill) and verbal (strong 3-2-1 countdown to the upcoming change in activity) instructions on the target speed were given. Visual instructions included a red line on the screen, or each participant's individual target speed, and a green line indicating their actual speed. Participants were required to match their current speed with the target speed as closely as possible for all of the six speed zones. Verbal cues were given by the same operator to reduce variability. Data was sampled at 50 Hz and exported to customized software to analyze the mean total work completed across each half of the protocol. The first half was activity performed with the first 12-min of the protocol and the second half was activity post the 13-min mark. Minute 12 to 13 was a passive resting period and consequently removed from analysis. The average mean and peak power was calculated for all sprints in each half. The average mean and peak power was also calculated for each of the first and third set of repeated sprints. All sprints were defined as the first occurrence of speed at 1 m·s^−1^ and from this point, a 4 s period was subsequently calculated (Serpiello et al., 2011). Peak power was determined as the highest single value recorded during a sprint. The coefficient of variation (CV) for mean power and mean peak power was 3.3 and 4.8%, respectively.

### Physiological measures

Blood oxygen saturation (S_p_O_2_) was continuously measured at the earlobe by pulse oximetry (SPO Medical equipment, Israel) and collected for each minute across the entire protocol. Average S_p_O_2_ data was analyzed for the first and second half of the protocol. Heart rate (HR) was recorded each minute of the protocol and averaged for the first plus second half. Rating of perceived exertion (RPE) was collected each minute, across both halves, via the Borg 6–20 scale. The mean RPE for each half of the protocol was analyzed.

Oxygenation of the vastus lateralis was monitored during all trials using a NIRS device (Oxymon MkIII, Artinis, The Netherlands). The emitter and detector probe pair, with an optode distance of 4 cm, were attached using black plastic spacers. Probes were placed on the muscle belly (~15 cm above the proximal border of the patella and 5 cm lateral to the midline of the thigh) of the dominant lower limb and protected from external light using black bandages. A modified form of the Beer–Lambert Law calculated micromolar changes in tissue oxyhaemoglobin [O_2_Hb] and deoxyhaemoglobin [HHb] across time using received optical densities from two continuous wavelengths of NIR light (763 and 855 nM). The differential optical path-length factor was 4.95 (Billaut and Buchheit, 2013). Skinfold thickness (5.7 ± 1.4 mm) was less than half of the emitter-detector distance in each participant.

Analysis concentrated on Δ[HHb], as it is primarily independent of total hemoglobin (De Blasi et al., 1993). Maximal [HHb] was obtained post-exercise via pressure cuff inflation (~300 mmHg), at the base of the thigh until the increased Δ[HHb] plateaued. The leg ischemia was performed whilst standing to mimic the sprint position. All Δ[HHb] values presented are expressed as a percentage of the amplitude of change between baseline and maximal [HHb] values. Data was acquired at 10 Hz throughout the entire protocol and averaged for each minute of the protocol. Data was also obtained during the recovery periods from repeated sprint efforts, within the first and third set of repeated sprints.

### Statistical analysis

Separate analyses were performed for each of the measures derived from the non-motorized and physiological variables. Each analysis was performed with the same mixed model using the general linear mixed-model procedure (Proc Mixed) in the Statistical Analysis System (version 9.4, SAS Institute, Cary NC). The fixed effects in the model were altitude (three levels: sea-level, 2,000, 3,000 m), the interaction of altitude with a dummy variable representing the second half of the testing session (to estimate mean change between the first and second half, representing mean fatigue in the second half), and a dummy variable representing the second trial at altitude (to adjust the altitude effects to the first trial at altitude). The following random effects were specified: participant identity (to allow for repeated measurement on participants) and its interaction with the second-half dummy variable (with unstructured covariance, to allow for individual second-half means, representing individual differences in fatigue); participant identity interacted with trial identity (to allow for the repeated measurement on participants within trials); participant identity interacted with a dummy variable representing non-sea level (to estimate individual responses to altitude); and the residual (representing error of measurement from half to half). There were insufficient observations to allow successful specification of different but correlated individual responses to the two altitudes.

All measures, except for the physiological measures, were log-transformed before analysis then back-transformed to express the changes in percent units. The changes were also expressed in standardized units for assessment of magnitude. Standardization was performed by dividing the change score of the log-transformed measure by the between-participant standard deviation derived from the random effects for participant and its interaction with trial identity; this between-participant standard deviation represents the differences between participant free of residual (measurement) error. Uncertainty in the changes was expressed as 90% confidence intervals and interpreted via the non-clinical magnitude-based inference approach (Hopkins et al., 2009). Standardized changes of 0.20, 0.60, 1.20, 2.0, and 4.0 were thresholds for small, moderate, large, very large, and extremely large effects, respectively (Hopkins et al., 2009). When the confidence interval for a change included small positive and negative effects, the change was deemed unclear. For clear effects, the likelihood that the true effect was substantial was indicated with the following scale: *possibly* (25–75%), *likely* (75 to <95%), *very likely* (95–99.5%), and *most likely* (>99.5%).

## Results

**Figure 2:**
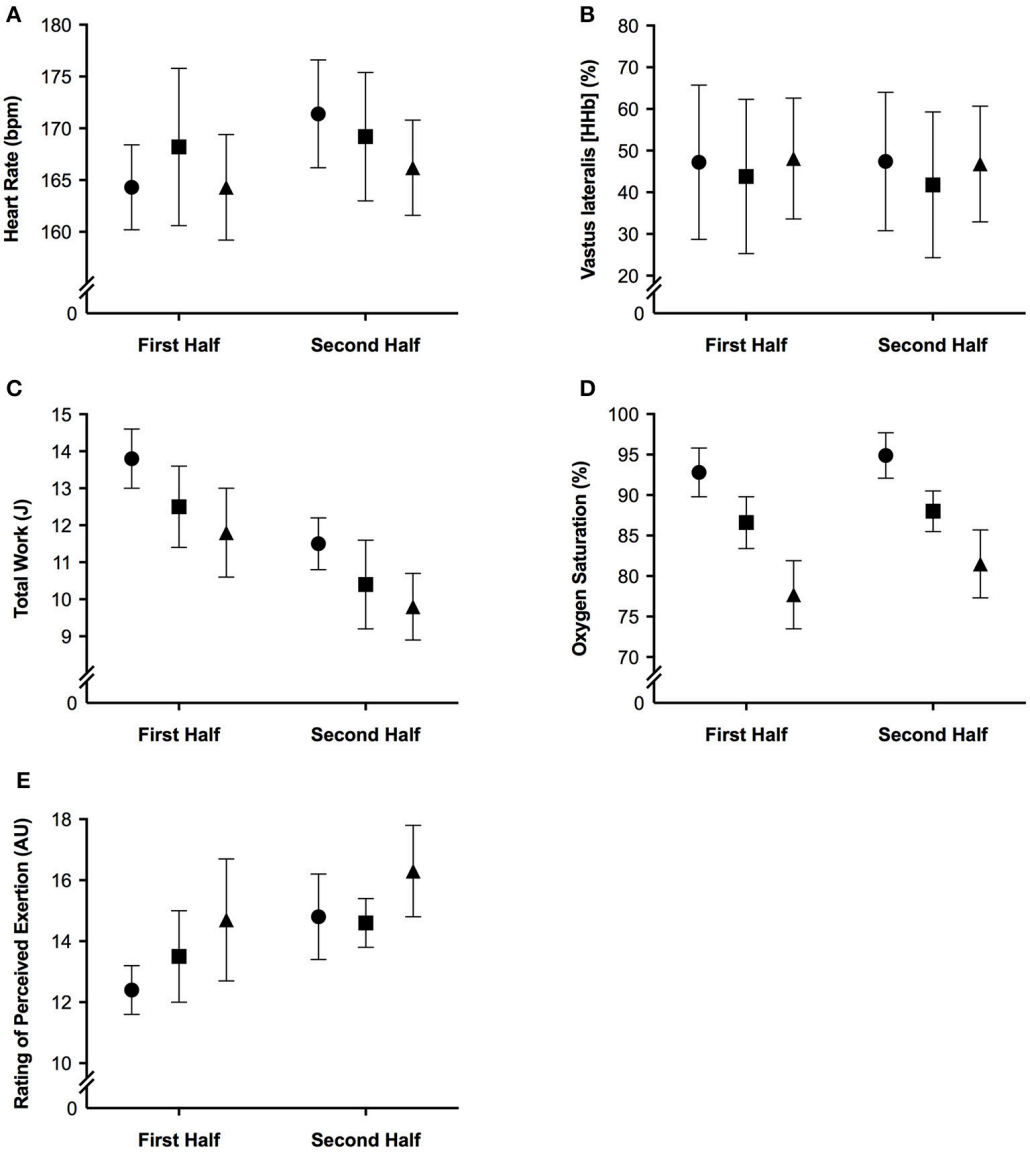
**The mean ± standard deviation for (A)** heart rate; **(B)** vastus lateralis deoxyhemoglobin; **(C)** total work; **(D)** oxygen saturation, and **(E)** rating of perceived exertion per half of the simulated team-sport running protocol. Circles: 0 m; rectangles: 2,000 m; triangles: 3,000 m.

**Figure 3:**
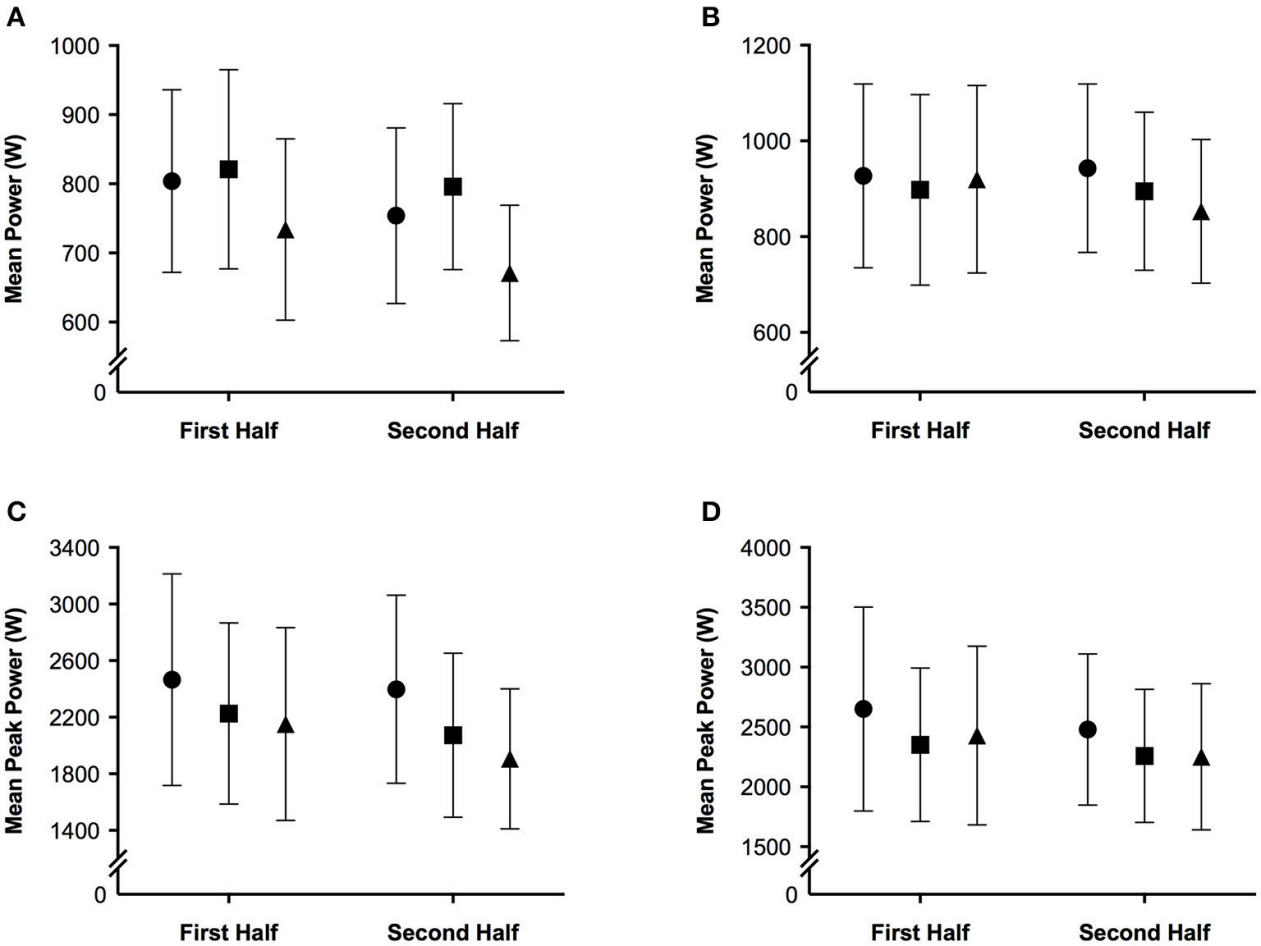
**The mean ± standard deviation for (A)** mean power during all sprint efforts; **(B)** mean power during repeated sprints only; **(C)** mean peak power during all sprint efforts; **(D)** mean peak power during repeated sprints only per half of the simulated team-sport running protocol. Circles: 0 m; rectangles: 2,000 m; triangles: 3,000 m.

**Figure 4:**
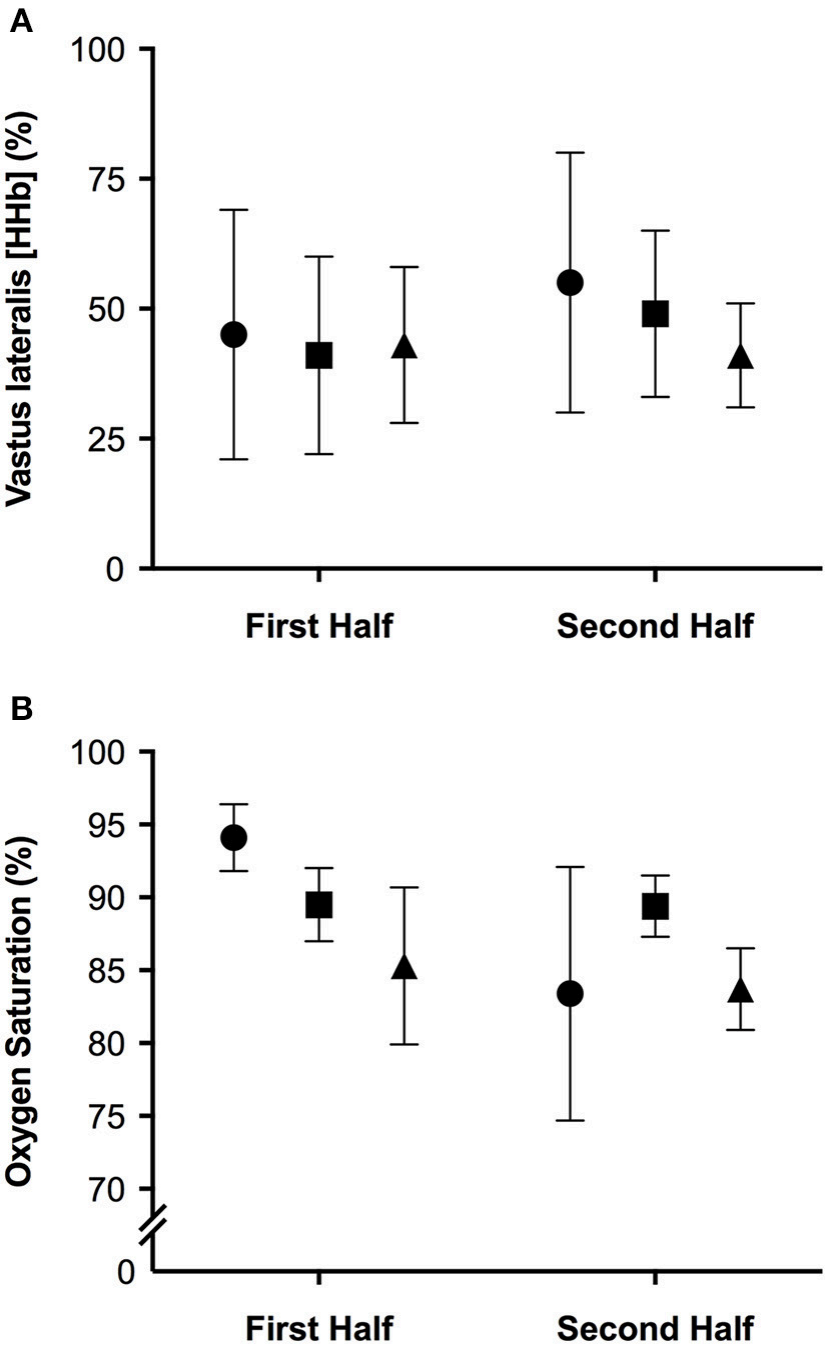
**The mean ± standard deviation values for (A)** vastus lateralis deoxyhemoglobin during recovery from repeated sprints; **(B)** oxygen saturation during repeated sprints, per half. Circles: 0 m; rectangles: 2,000 m; triangles: 3,000 m.

The mean and ± standard deviation of mean heart rate, vastus lateralis deoxyhemoglobin, total work, oxygen saturation, and rating of perceived exertion per half of the simulated team-sport running protocol at each altitude are presented in Figure 2. The mean ± standard deviation for mean and peak power during all sprint efforts, per level of altitude and each half, is presented in Figure 3. The mean ± standard deviation for Vastus lateralis [HHb] during recovery periods and average blood oxygenation saturation per half is presented in Figure 4. The magnitude of change in performance and physiological variables, across the entire protocol, per half, and compared across levels of altitude are presented in Table 1. The magnitude of change in performance and physiological variables, during repeated sprints, per half, and compared across levels of altitude are presented in Table 2. The magnitude of change in performance and physiological variables at altitude, across the second half of the protocol, and during the final trial, are presented in Table 3. The magnitude of change in performance and physiological variables at altitude, during repeated-sprints only, across the second half of the protocol, and during the final trial, are presented in Table 4.

**Table 1:** **The difference in magnitude of change between levels of altitude on mean total work, heart rate, vastus lateralis deoxygenation, rating of perceived exertion, blood oxygenation saturation, mean sprint power, and mean sprint peak power across the entire protocol**.

	**Comparison (m)**	**Difference across entire protocol**	**Difference in second half of protocol**
Mean total work (%)	2,000–0	−10.3, ±5.5; ↓^***^	**0.1, ±4.7**
	3,000–0	−14.9, ±5.2; ↓^****^	**−0.3, ±4.7**
	3,000–2,000	−5.1, ±2.7; ↓^***^	**−0.4, ±4.7**
Mean HR (bpm)	2,000–0	0.4, ±2.5	−3.6, ±2.3
	3,000–0	−2.7, ±2.4; ↓^**^	−3.4, ±2.4; ↓^**^
	3,000–2,000	−3.1, ±2.5; ↓^**^	**0.2, ±2.5**
Mean Δ[HHb] (%)	2,000–0	−3.9, ±9.1	−2.2, ±3.3; ↔^00^
	3,000–0	0.7, ±8.8	−1.5, ±3.3; ↔^00^
	3,000–2,000	4.5, ±7.3; ↑^*^	0.7, ±3.3; ↔^00^
Mean RPE (AU)	2,000–0	0.1, ±1.6	−1.1, ±0.8; ↓^**^
	3,000–0	1.7, ±1.4; ↑^**^	−0.5, ±0.8
	3,000–2,000	1.5, ±1.3; ↑^**^	0.6, ±0.8; ↑^*^
Mean S_p_O_2_(%)	2,000–0	−7.7, ±2.5; ↓^****^	1.4, ±1.4; ↑^**^
	3,000–0	−14.6, ±2.4; ↓^****^	2.3, ±1.3; ↑^***^
	3,000–2,000	−7.0, ±1.7; ↓^****^	0.9, ±1.0; ↑^*^
Sprint mean Power (%)	2,000–0	−1.9, ±4.9; ↔^00^	3.8, ±6.8; ↑^*^
	3,000–0 m	−13.8, ±4.3; ↓^****^	−1.9, ±3.6; ↔^00^
	3,000–2,000	−12.0, ±2.9; ↓^****^	−5.5, ±3.5; ↓^**^
Sprint mean peak Power (%)	2,000–0	−10.1, ±6.4; ↓^**^	−4.9, ±6.1; ↓^*^
	3,000–0	−15.6, ±6.0; ↓^***^	−8.7, ±5.8; ↓^*^
	3,000–2,000	−6.1, ±3.7; ↓^*^	**−4.0, ±6.1; ↔^00^**

Values are presented as raw or % change in mean, ±90% CI or ±99% CI; Direction of response: positive ↑ negative ↓ trivial ↔; Symbols denote:

* possibly,

** likely,

*** very likely, and

**** *most likely chance of the true effect exceeding a small (0.2) effect. Trivial changes indicated by ^0^. m, meters; HR, heart rate; Δ[HHb], change in deoxyhemoglobin; RPE, rating of perceived exertion; S_p_O_2_, blood oxygenation saturation. Bold text indicates change in the mean at the 99% confidence interval (CI)*.

**Table 2:** **The difference in magnitude of change between levels of altitude on mean and peak power, vastus lateralis deoxygenation during recovery periods, and blood oxygenation saturation during repeated sprints only**.

	**Comparison**	**Difference across entire protocol**	**Difference in second half of protocol**
Repeated sprint mean power (%)	2,000–0	−4.6, ±3.3; ↓^*^	−1.7, ±5.3;↔^00^
	3,000–0	−5.5, ±3.2; ↓^*^	−8.8, ±4.9; ↓^**^
	3,000–2,000	**−1.0, ±1.8; ↔^0000^**	−7.2, ±5.0; ↓^*^
Repeated sprint mean peak power (%)	2,000–0	−7.8, ±7.3; ↓^*^	1.3, ±12.6
	3,000–0	−7.5, ±7.2; ↓^*^	−2.2, ±12.1
	3,000–2,000	−0.3, ±5.1; ↔^0000^	−3.5, ±12.0
Mean recovery HHb (%)	2,000–0	−3.5, ±11.6	−1.3, ±12.3
	3,000–0	−6.9, ±10.7; ↓^*^	−11.0, ±12.3; ↓^**^
	3,000–2,000	−3.4, ±12.2	−9.7, ±12.3; ↓^**^
Mean set S_p_O_2_(%)	2,000–0	−0.4, ±6.8	15.0, ±3.7; ↑^****^
	3,000–0	−5.3, ±6.5; ↓^**^	13.1, ±3.6; ↑^****^
	3,000–2,000	−4.9, ±6.4	−1.9, ±3.5

Values are presented as % change in mean, ± 90% CI or ±99% CI; Direction of response: positive ↑ negative↓ trivial ↔; Symbols denote:

* possibly,

** likely,

^***^very likely, and

**** *most likely chance of the true effect exceeding a small (0.2) effect. Trivial changes indicated by ^0^. m, meters; Δ[HHb], change in deoxyhemoglobin; S_p_O_2_, blood oxygenation saturation. Bold text indicates change in the mean at the 99% confidence interval (CI)*.

**Table 3:** **The difference in magnitude of change at altitude on mean total work, heart rate, vastus lateralis deoxygenation, rating of perceived exertion, blood oxygenation saturation, mean sprint power, and mean sprint peak power across the second half of the protocol and during the final trial, irrespective of altitude**.

	**Altitude (m)**	**Difference in second half of protocol**	**Difference in final trial**
Mean total work (%)	0	−16.9, ±3.5; ↓^****^	−0.5, ±2.9
	2,000	−16.8, ±3.5; ↓^****^	
	3,000	−17.2, ±3.5; ↓^****^	
Mean HR (bpm)	0	4.5, ±2.7; ↑^**^	−0.2, ±2.4
	2,000	0.7, ±2.5; ↔^00^	
	3,000	0.9, ±2.6	
Mean Δ[HHb] (%)	0	0.2, ±2.8	−1.1, ±7.2
	2,000	−2.0, ±2.7; ↓^*^	
	3,000	−1.3, ±2.7; ↓^*^	
Mean RPE (AU)	0	2.2, ±1.1; ↑^*^	0.2, ±1.3
	2,000	**1.1, ±1.1; ↔^00^**	
	3,000	1.7, ±1.1; ↑^*^	
Mean S_p_O_2_(%)	0	**0.5, ±1.1; ↔^000^**	−0.6, ±1.7; ↑^*^
	2,000	1.9, ±0.8; ↑^***^	
	3,000	2.8, ±0.8; ↑^****^	
Sprint mean power (%)	0	−6.3, ±2.9; ↓^****^	10.9, ±3.7; ↑^****^
	2,000	−2.8, ±3.0; ↓^**^	
	3,000	−8.1, ±2.8; ↓^****^	
Sprint mean peak power (%)	0	−1.8, ±8.4	−2.3, ±6.2
	2,000	−6.5, ±8.0	
	3,000	−10.3, ±7.6; ↓^***^	

Values are presented as raw or % change in mean, ±90% CI or ± 99% CI; Direction of response: positive ↑ negative↓ trivial ↔; Symbols denote:

* possibly,

** likely,

*** very likely, and

**** *most likely chance of the true effect exceeding a small (0.2) effect. Trivial changes indicated by ^0^. m, meters; HR, heart rate; Δ[HHb], change in deoxyhemoglobin; RPE, rating of perceived exertion; S_p_O_2_, blood oxygenation saturation. Bold text indicates change in the mean at the 99% confidence interval (CI)*.

**Table 4:** **The difference in magnitude of change at altitude on mean and peak power, vastus lateralis deoxygenation during recovery periods, and blood oxygenation saturation during repeated sprints only and during the final trial, irrespective of altitude**.

	**Altitude (m)**	**Difference in second half of protocol**	**Difference in final trial**
Repeated sprint mean power (%)	0	2.4, ±8.3	**0.8, ±1.8; ↔^0000^**
	2,000	0.6, ±8.2	
	3,000	−6.6, ±7.6; ↓^*^	
Repeated sprint mean peak power (%)	0	−4.3, ±10.6; ↓^*^	**−3.0, ±4.9; ↔^00^**
	2,000	−3.1, ±10.8	
	3,000	−6.4, ±10.4; ↓^*^	
Mean recovery HHb (%)	0	9.8, ±7.9; ↑^**^	−2.0, ±12.2
	2,000	8.5, ±7.9; ↑^**^	
	3,000	−1.3, ±7.9	
Mean set S_p_O_2_(%)	0	−14.7, ±2.6; ↓^****^	−2.1, ±6.4
	2,000	0.3, ±2.3	
	3,000	−1.5, ±2.6; ↓^*^	

Values are presented as % change in mean, ±90% CI or ± 99% CI; Direction of response: positive ↑ negative↓ trivial ↔; Symbols denote:

* possibly,

** likely,

^***^very likely, and

**** *most likely chance of the true effect exceeding a small (0.2) effect. Trivial changes indicated by ^0^. m, meters; Δ[HHb], change in deoxyhemoglobin; S_p_O_2_, blood oxygenation saturation. Bold text indicates change in the mean at the 99% confidence interval (CI)*.

## Discussion

Mean total work was substantially reduced during simulated team-sport movement at 2,000 and 3,000 m compared to sea-level. Mean total work was also most likely reduced in the second half of the protocol compared to the first at sea-level, 2,000 and 3,000 m. During sprint efforts across the simulated running protocol, mean and peak power was reduced at 3,000 m compared to sea-level. During repeated-sprints in the second half of the protocol, mean power was possibly reduced at 3,000 m, with no clear differences at 2,000 m or sea-level. During recovery from repeated-sprint efforts, vastus lateralis [HHb] was possibly reduced at 3,000 m compared to SL. This study is the first to use linear mixed modeling to examine the effect of titrated altitude on muscle oxygenation during simulated team-sport movement. This statistical approach provides detail on the percentage effects of fixed and individual factors, superior to other commonly used statistical methods, that may contribute to a decrement in performance at altitude.

During repeated-sprint efforts, vastus lateralis [HHb] was likely higher in the second half at 2,000 m compared SL. This reoxygenation impairment is in agreement with the increased [HHb] (relative to normoxia) and performance decrement observed during recovery of ten 10 s sprints under 0.13 F_i_O_2_ Billaut and Buchheit (2013) during occlusion-free conditions, it is still debated whether the primary determinant of muscle post-exercise reoxygenation is muscle O_2_ delivery or local utilization (Hamaoka et al., 1998; Kime et al., 2003). In the present study, the incomplete muscle reoxygenation observed in the second half at 3,000 m was likely related to limited muscle O_2_ delivery post-sprint (Billaut and Buchheit, 2013). The resynthesis of PCr is derived solely from aerobic sources (Harris et al., 1976). In hypoxia, aerobic capacity is considerably impaired (Gore et al., 1996) and an inverse relationship between O_2_ supply and PCr resynthesis rate has been established *in vitro* (Idstrom et al., 1985). In normoxia, PCr stores recovered to only 45% of resting concentrations after five 6 s sprints interspersed with 24-s passive recovery (Dawson et al., 1997). The current study employed 4-s repeated-sprints interspersed with 14-s recovery. Between RS efforts, PCr was likely not fully resynthesized due to a hypoxia-induced decrease in O_2_ availability to working musculature, with S_p_O_2_ 7.7 and 14.6% lower across the entire protocol at 2,000 and 3,000 m, respectively. Metabolic recovery and subsequent sprint performance was likely compromised, although the resynthesis of PCr was not measured in the present study. Impaired O_2_ transport in hypoxia likely increased the contribution of anaerobic glycolysis to exercise metabolism, (Morales-Alamo et al., 2012) accelerating the development of peripheral muscle fatigue and decreased central motor drive (Amann and Dempsey, 2008). Cerebral oxygenation and electromyography data would provide a detailed understanding of the acute fatigue mechanisms during repeated-sprint sets at titrated altitudes.

Mean total work performed during the simulated team-sport movement was considerably diminished at 2,000 and 3,000 m compared to SL. This is in agreement with the large reduction in total work during three sets of (9 × 4 s) repeated running sprints at 2,000 and 3,000 m simulated altitude (Goods et al., 2014). In the present study, total work was reduced by ~5.1% per 1,000 m rise in altitude, lower than the ~7% per 1,000 m decline during 5 min time-trial performance (Clark et al., 2007), and 14% per 1,000 m decrement in a time-to-exhaustion (total duration of 308 s) test (Wehrlin and Hallen, 2006). The smaller reduction in the present study may be attributed to variation in the length and type of exercise. In contrast with the relatively short-duration, all-out time-trial (Clark et al., 2007), and time-to-exhaustion (Wehrlin and Hallen, 2006) tests, the 26.4 min protocol included lower-intensity movements and recovery, closer to replicating team-sport running. The recovery afforded by these lower-intensity periods (and associated lower energy expenditure) likely assisted the maintenance of repeated-sprint performance. Although, an active recovery from short repeated cycling sprints may disrupt the resynthesis of PCr (Spencer et al., 2008), our findings are reinforced by data collected during an elite youth soccer match at 1,600 m (Garvican et al., 2013). High-speed running was impaired by 15% and low-speed running plus total distance were reduced by 9 and 8%, respectively (Garvican et al., 2013). During a match at 3,600 m, peak 5 min running distance by elite youth soccer athletes was also reduced by 13–16% (Aughey et al., 2013). Collectively, this data demonstrates the negative impact of altitude on team-sport running performance.

Participants may have paced their effort, via self-selecting a lower intensity under hypoxia, between repeated-sprint sets in an attempt to preserve the ability to perform high-intensity efforts. Whilst the majority of pacing research focuses on continuous events (de Koning et al., 2011), pacing is also evident during repeated-sprint exercise. Knowledge of an exercise end-point can determine athletic performance. During a deception trial (where participants were uninformed on the number of sprints to be completed), less muscle mass was recruited and mechanical work lowered, presumably to avoid excessive metabolic disturbances, and muscle fatigue (Billaut et al., 2011). In the present study, participants were aware of the duration and number of sprints to be completed. Participants were also visually guided on the speed to be performed throughout the entire protocol, including the sprints.Participants may have been slower to reach target velocities or maintain these velocities during sprint efforts embedded in the protocol, in the aim of maintaining repeated-sprints located at the end of the protocol. The preservation of repeated-sprint efforts at 2,000 m are in agreement with the maintenance of repeated-running sprints in hypoxia from 12 to 21% O_2_ (Bowtell et al., 2014). Fatigue index (calculated as the percentage decrease in peak speed from fastest to slowest speed) was greater when breathing 12% O_2_ (corresponding altitude of 4,345 m; Bowtell et al., 2014). At a simulated altitude of 4,000 m, mean power output was also reduced during the first set of 9 × 4 s running repeated-sprint (Goods et al., 2014). Mean power was also lowered in subsequent sets at 2,000, 3,000, and 4,000 m (Goods et al., 2014), emphasizing the critical role of O_2_ in PCr resynthesis and repeated-sprint performance (Harris et al., 1976).

Due to the chaotic nature of competition, athletes are unaware of the number of high-intensity bouts to be completed. Many high-intensity actions are performed when the ball is in possession, dispute or an opportunity to score arises (Faude et al., 2012). Thus, team-sport athletes are less able to modulate lower speed activities to preserve sprint capacity. This is in opposition with laboratory-based repeated-sprint studies, where participants are typically aware of the number of efforts to be completed. In the present study, participants were informed about lower-intensity activities and despite constant visual information on the speed to be maintained, they were unable or chose not to do so in hypoxia. At 3,000 m simulated altitude, the hypoxic stress appears to negatively influence physical output compared to 2,000 m. Coaching and conditioning staff should be aware of the negative impact of 3,000 m altitude upon team-sport running performance. Athletes may need to be interchanged or substituted when competing at 3,000 m, to minimize the detrimental impact of high altitude and allow sufficient recovery from high-speed running. Training at 3,000 m prior to competing at altitude may negate the detrimental effects of hypoxia on team-sport running, although this remains to be investigated. The short protocol duration of 26.4 min, in the present study, was designed to simulate an intensified period of team-sport activity. A 30 min simulated team-sport running protocol is a reliable tool for assessing and monitoring the physiological and performance of team-sport activity (Sirotic and Coutts, 2008). Increasing the protocol duration of the current study to 52.8 or 79.2 min could more closely replicate the duration of a team-sport match although it is unclear what impact this would have on test-retest variation, particularly at hypoxia. Future studies should investigate the impact of hypoxia on a longer simulated running protocol and use a linear-mixed modeling approach to estimate the individual and fixed factors that contribute to performance. Future studies investigating individual responses to simulated altitude should also monitor the training loads, sleep, nutrition, and recovery of participants during performance testing.

## Conclusion

During simulated team-sport movement, repeated-sprint and single-sprint efforts are compromised at 3,000 m altitude, possibly due to limited muscle O_2_ availability during recovery periods. To overcome this decrement, participants reduced their total work completed despite strong verbal encouragement. Whilst this strategy may have assisted in largely maintaining repeated-sprint and single-sprint efforts at 2,000 m, the elevated physiological demands at 3,000 m may have been overwhelming. Consequently, repeated-sprint and single-sprint performance was subsequently impaired. Under hypoxia, the decrement in work completed during intermittent running is lower than previously reported for time-trial and time-to-exhaustion tests.

## Ethics statement

This study was carried out in accordance with the recommendations of ethical guidelines, as declared by the Victoria University High Risk Ethics Committee, with written informed consent from all subjects. All subjects gave written informed consent in accordance with the Declaration of Helsinki. The protocol was approved by the Victoria University High Risk Ethics Committee.

## Author contributions

Conceived and designed the experiments: FB and RA. Performed experiments: AS, FB, RR, MV, and RA. Analyzed data: AS. Interpreted results of research: AS, RA, RR, MV, FB, WH. Drafted manuscript and prepared tables/figures: AS. Edited, critically revised paper, and approved final version of manuscript: AS, RR, MV, FB, WH, and RA.

### Conflict of interest statement

The authors declare that the research was conducted in the absence of any commercial or financial relationships that could be construed as a potential conflict of interest. The handling Editor declared a past co-authorship with several of the authors FB and RA and states that the process nevertheless met the standards of a fair and objective review.
